# Effects of neuraxial or general anaesthesia on postoperative adverse events in oldest-old patients (aged 90 years and older) with intertrochanteric fractures: a retrospective study

**DOI:** 10.1186/s12891-023-06973-y

**Published:** 2023-10-23

**Authors:** Wei-dong Guo, Yue Li, Jia-hui Li, Feng Han, Guo-shun Huang

**Affiliations:** 1 Department of Orthopedic Surgery, General Hospital of Tisco, Yingxin Road 7#, Jiancaoping District, Taiyuan City, Shanxi Province 030008 China; 2 Shanxi Medical University, Xinjian South Road 56#, Yingze District, Taiyuan City, Shanxi Province 030607 China

**Keywords:** Anaesthesia, Adverse event, Oldest-old, Intertrochanteric fracture

## Abstract

**Background:**

To retrospectively analyse postoperative adverse events in oldest-old patients (aged 90 years and older) with intertrochanteric fractures treated under various anaesthetic techniques.

**Methods:**

A total of 153 consecutive patients participated in this study, of which 127 patients who underwent surgery with neuraxial anaesthesia or general anaesthesia for intertrochanteric fractures between October 2019 and October 2022 were eligible and evaluated. They were divided into the neuraxial anaesthesia and general anaesthesia groups. The demographic characteristics and postoperative adverse events were compared between the two groups.

**Results:**

A total of 13 patients (10.24%), including 6 in the neuraxial anaesthesia group (8.22%) and 7 in the general anaesthesia group (12.96%), died within 30 days after surgery. No significant differences between the two groups were observed. Postoperative delirium occurred in 40 patients (31.49%), including 17 (23.29%) in the neuraxial anaesthesia group and 23 (42.59%) in the general anaesthesia group; there was a significant difference between the two groups [P = 0.02, odds ratio (OR) = 0.41]. The other postoperative adverse events, including heart failure, acute stroke, acute myocardial infarction, pulmonary disease, anaemia, deep vein thrombosis, hypoproteinaemia, and electrolyte disorders, were not significantly different between the two groups.

**Conclusion:**

Our data suggest that different anaesthesia methods do not affect the incidence of adverse events, such as death within 30 days after surgery in oldest-old patients with intertrochanteric fractures. However, more patients developed delirium after surgery in the general anaesthesia group (23, 42.59%) than in the neuraxial anaesthesia group (17, 23.29%); this may indicate that spinal anaesthesia reduces the incidence of postoperative delirium (P = 0.02, OR = 0.41).

**Trial registration:**

Retrospectively registered.

## Background

Hip fracture is a major health concern in the ageing population; over 7 million older adults globally are estimated to develop hip fractures annually, with intertrochanteric fractures (IFs) accounting for approximately 60–70% [1, 2]. Notably, most patients with hip fractures undergo surgery. The proximal femoral nail antirotation (PFNA) technique is an effective minimally invasive procedure to treat IFs [3], and the most commonly used anaesthesia types include neuraxial anaesthesia (NA) and general anaesthesia (GA) [4]. Determining the most efficient anaesthetic technique for older patients with hip fractures and how these techniques affect their postoperative outcomes has remained controversial [5, 6]. GA increases the risk of death, delirium, and other postoperative complications [4]; however, NA leads to low back pain and psychological stress, negatively affecting patients [7].

People aged 90 years and older are often referred to as the oldest-old [8], and because of their vulnerability and high perioperative mortality, they become a special population for surgical procedures [9]. With the increase in global ageing, the oldest-old population is increasing. Previous studies have suggested that a surgical delay of > 24 h, coexisting diabetes, or cardiovascular disease are the primary causes of postoperative mortality in elderly patients with hip fractures [6], and surgical treatment of hip fractures is significantly challenging in oldest-old patients. Due to concomitant factors such as frailty and comorbidities, there is a significantly higher prevalence of perioperative complications and mortality in the oldest-old patients with hip fractures than those aged ≤ 89 [9]. However, previous studies on the oldest-old patients with hip fractures have rarely focused on anaesthetic modalities.

Based on the current research status, we hypothesised that the anaesthesia type would not influence the incidence of postoperative adverse events in the oldest-old patients with IFs undergoing surgery with PFNA implantation. Therefore, we retrospectively analysed the oldest-old patients with IFs who underwent treatment with PFNA implantation at our centre and evaluated the outcomes of the anaesthesia methods. To our knowledge, this is the first study to discuss surgical anaesthesia in oldest-old patients with hip fractures.

## Methods

### Study design and participants

This study is a retrospective study. A total of 153 consecutive patients were enrolled in the study, and 127 eligible patients were evaluated. The inclusion criteria were: (1) aged 90 years and older and (2) diagnosed with IF and treated with PFNA implantation. Exclusion criteria were as follows: (1) history of hip surgery, (2) multiple fractures, (3) open fractures, and (4) pathological fractures. This study was conducted at our institution in accordance with the ‘WMA Declaration of Helsinki 2013–Ethical Principles for Medical Research Involving Human Subjects’. This study was approved by the Ethics Committee of the General Hospital of Tisco (ID: KY202302), and the requirement for informed consent was waived. All regulations and guidelines were followed while performing the experiments.

### Assessment criteria

The patients included in the study were divided into the GA and NA groups according to the types of anaesthesia received. The patient characteristics included age, sex, body mass index (BMI), time from fracture to surgery, fracture type, length of hospital stay, comorbidities, American Society of Anesthesiologists (ASA) grade, operation time, intraoperative blood loss, intensive care unit (ICU) stay after surgery, and return to residence after discharge. The fracture type was assessed using the EVANS classification [10]. Comorbidities were assessed using the Charlson comorbidity scale [11].

Postoperative adverse events included heart failure, acute stroke, acute myocardial infarction, pulmonary disease, anaemia, deep vein thrombosis, delirium, hypoproteinaemia, electrolyte disorders, and death within 30 days of surgery. The following factors should be considered to diagnose acute myocardial infarction, heart failure, acute stroke, or deep vein thrombosis: clinical symptoms, medical history, laboratory tests, and additional tests, if necessary. Furthermore, pneumonia was confirmed based on the clinical and radiological findings when patients developed lung infections during hospitalisation. Delirium was assessed using the 3D-CAM scale [12], and haemoglobin levels < 70 g/L indicated anaemia. Hypoalbuminaemia was defined as serum albumin levels < 30 g/L, and hyponatraemia was defined as serum sodium levels > 135 mmol/L. Hypokalaemia was defined as a serum potassium levels < 3.5 mmol/L.

A multidisciplinary geriatric orthopaedic team managed elderly patients with fractures at our centre, and ward rounds were conducted twice daily. The team included a respiratory physician, cardiologist, gastroenterologist, intensivist, and the three orthopaedic surgeons responsible for the procedure. Adverse events were adjudicated by a multidisciplinary geriatric orthopaedic team and two independent orthopaedic surgeons.

### Anaesthesia procedures

Patients in the NA group received combined spinal-epidural anaesthesia. For patients undergoing NA, preoperative routine fluid replacement was rapidly expanded, and the total amount of fluid replacement was 10 mL/kg body weight. Fluid replacement was completed within 30 min to maintain stable intraoperative circulation. The patient was placed in the lateral decubitus position with the affected limb on top. Combined spinal–epidural anaesthesia was performed in the L3–L4 or L4–L5 intervertebral spaces. After successful epidural puncture, a lumbar puncture needle was inserted, guided by an epidural puncture needle. This was then passed through the epidural puncture needle port and punctured the arachnoid membrane. Then, the stylet was withdrawn once cerebrospinal fluid could be seen flowing out of the needle. Immediately, 5–7 mg ropivacaine mixed with cerebrospinal fluid or normal saline was injected in the cephalad direction. The lumbar needle was withdrawn, and an epidural catheter was placed through the epidural needle. The catheter (3 cm) was left in the epidural space. The epidural needle was withdrawn and the puncture site was covered with sterile gauze to secure the catheter. The plane of anaesthesia was controlled below T10. Blood pressure fluctuations were controlled within 10%. The criteria for achieving satisfactory anaesthetic results were no pain in the affected limb and minor fluctuations in blood pressure during mobilisation. If pain developed during surgery, a small volume of local anaesthetic agent was administered into the epidural space. Simultaneously, appropriate vasopressors were pumped during the operation to maintain perioperative hemodynamic stability.

For patients undergoing GA, tracheal intubation was induced with etomidate at 0.2 mg/kg, dexmedetomidine at 1 mg/kg pumped for 10–15 min, sufentanil at 0.25 mg/kg, and cisatracurium at 0.3 mg/kg. Respiratory rate was maintained at 12–18 breaths/min and tidal volume at 8 mL/kg on mechanical ventilation. The maintenance period was perpetuated with propofol (6 mg/kg/h) and remifentanil (8 µg/kg/h); muscle relaxation was maintained with cisatracurium (0.1 mg/kg/h). Sufentanil was intermittently administered as appropriate during the operation.

### Statistical analysis

SPSS version 21.0 was used to analyse the collected data. Normally distributed or approximately normally distributed data are presented as means and standard deviations, and non-normally distributed data are presented as medians and interquartile ranges. Categorical variables are presented as numbers and percentages. Student’s t-test or Mann–Whitney U test was used to compare continuous data between groups. For categorical data, χ^2^ test or Fisher exact test was used. Statistical significance was set at P < 0.05.

## Results

We reviewed 127 oldest-old patients with IFs treated with PFNA implantation. The NA and GA groups had median ages of 92. Regarding postoperative ICU admission, 23 patients (31.51%) in the NA group and 21 (38.89%) in the GA group entered the ICU after surgery, with no statistically significant difference between the two groups. Simultaneously, 45 patients (61.64%) in the NA group and 37 patients (68.52%) in the GA group returned to their residences after discharge, with no statistically significant difference between the two groups. The other demographic and clinical characteristics (Table 1) were not significantly different between the two groups.

**Table 1:** Demographic and clinical characteristics of the patients

	Total (n = 127)	NA (73)	GA (54)	T/Z/χ2	P-Value
Sex, n (%)				0.19	0.67
Male	56 (44.09%)	31 (42.47%)	25 (46.30%)		
Female	71 (55.91%)	42 (57.53%)	29 (53.70%)		
Age, median (P25–P75) (years)	92 (91–93)	92 (91–93)	91 (91–93)	-1.85	0.06
BMI, mean ± SD (kg/m^2^)	19.95 ± 2.54	20.07 ± 2.54	19.80 ± 2.57	0.59	0.55
Charlson Comorbidity Scale, n (%)				0.11	0.74
<6	85 (66.93%)	48 (65.75%)	37 (68.52%)		
≥6	42 (33.07%)	25 (34.25%)	17 (31.48%)		
Fracture type, n (%)				-0.53	0.60
EVANS 1	10 (3.30%)	5 (1.92%)	5 (5.13%)		
EVANS 2	11 (4.40%)	7 (5.77%)	4 (2.56%)		
EVANS 3	44 (39.56%)	27 (42.31%)	17 (35.90%)		
EVANS 4	44 (40.66%)	25 (40.38%)	19 (41.03%)		
EVANS 5	18 (12.09%)	9 (9.62%)	9 (15.38%)		
ASA score, n (%)				-0.39	0.69
2	32 (21.98%)	17 (19.23%)	15 (25.64%)		
3	69 (62.64%)	41 (65.38%)	28 (58.97%)		
4	26 (15.38%)	15 (15.38%)	11 (15.38%)		
Fracture to operation, median (P25–P75) (days)	3 (2–4)	3 (2–4)	3 (2–4)	-0.17	0.87
Length of stay, mean ± SD (days)	12.17 ± 4.57	11.84 ± 4.27	12.61 ± 4.97	-0.94	0.35
Operation time, mean ± SD (min)	77.78 ± 26.86	79.19 ± 28.54	74.87 ± 24.53	0.69	0.49
Intraoperative blood loss, mean ± SD (ml)	107.95 ± 39.24	107.53 ± 38.87	108.52 ± 40.10	-0.14	0.89
Entering ICU after surgery, n (%)				0.75	0.39
Yes	44 (34.65%)	23 (31.51%)	21 (38.89%)		
No	83 (65.35%)	50 (68.49%)	33 (61.11%)		
Return to residence after discharge, n (%)				0.64	0.42
Yes	82 (64.57%)	45 (61.64%)	37 (68.52%)		
No	45 (35.43%)	28 (38.36%)	17 (31.48%)		

*P < 0.05, statistical significance

NA, neuraxial anaesthesia group; GA, general anaesthesia group; BMI, body mass index; ASA, American Society of Anesthesiologists; ICU, intensive care unit; SD, standard deviation

The incidences of postoperative adverse events are shown in Table 2. A total of 40 (31.49%) patients, including 17 (23.29%) in the NA group and 23 (42.59%) in the GA group, developed delirium after surgery; the difference between the two groups was statistically significant [P = 0.02, odds ratio (OR) = 0.41]. A total of 13 (10.24%) patients, including 6 (8.22%) in the NA group and 7 (12.96%) in the GA group, died within 30 days after surgery; there was no statistically significant difference between the two groups. The other postoperative adverse events did not show a statistically significant difference between the two groups.

**Table 2:** Postoperative adverse events

	Total (n = 127)	NA (73)	GA (54)	χ2	P-Value
Death within 30 days, n (%)				0.76	0.38
Yes	13 (10.24%)	6 (8.22%)	7 (12.96%)		
No	114 (89.76%)	67 (91.78%)	47 (87.04%)		
Heart Failure, n (%)				0.12	0.73
Yes	15 (11.81%)	8 (10.96%)	7 (12.96%)		
No	112 (88.19%)	65 (89.04%)	47 (87.04.%)		
Acute stroke, n (%)				0.36	0.54
Yes	14 (11.02%)	7 (9.59%)	7 (12.96%)		
No	113 (88.98%)	66 (90.41%)	47 (87.04%)		
Acute myocardial infarction, n (%)				0.71	0.40
Yes	11 (8.67%)	5 (6.85%)	6 (11.11%)		
No	116 (66.93%)	68 (93.15%)	48 (88.89%)		
Pneumonia, n (%)				0.11	0.74
Yes	38 (29.92%)	21 (28.77%)	17 (31.48%)		
No	82 (64.57%)	52 (71.23%)	37 (68.52%)		
Anaemia, n (%)				0.01	0.92
Yes	23 (18.11%)	13 (17.81%)	10 (18.52%)		
No	104 (81.89%)	60 (82.19%)	44 (81.48%)		
Deep vein thrombosis, n (%)				1.64	0.20
Yes	24 (18.90%)	11 (15.07%)	13 (24.07%)		
No	103 (81.10%)	62 (84.93%)	41 (75.93%)		
Delirium, n (%)				5.36	0.02^*^
Yes	40 (31.49%)	17 (23.29%)	23 (42.59%)		
No	87 (68.50%)	56 (76.71%)	31 (57.41%)		
Hypoproteinaemia, n (%)				0.63	0.43
Yes	56 (44.09%)	30 (41.10%)	26 (48.15%)		
No	71 (55.91%)	43 (58.90%)	28 (51.85%)		
Electrolyte disorder, n (%)				0.02	0.90
Yes	65 (51.18%)	37 (50.68%)	28 (51.85%)		
No	62 (48.82%)	36 (49.32%)	26 (48.15%)		

*P < 0.05, statistical significance

NA, neuraxial anaesthesia group; GA, general anaesthesia group

## Discussion

In this study, 127 oldest-old patients who underwent NA or GA for IFs were evaluated. A total of 13 (10.24%) patients, including 6 (8.22%) in the NA group and 7 (12.96%) in the GA group, died within 30 days after surgery; there was no statistically significant difference between the two groups. A total of 40 (31.49%) patients, including 17 (23.29%) in the NA group and 23 (42.59%) in the GA group, developed delirium after surgery; there was a significant difference between the two groups (P = 0.02, OR = 0.41). The other adverse events, including heart failure, acute stroke, acute myocardial infarction, lung disease, anaemia, deep vein thrombosis, hypoproteinaemia, and electrolyte imbalance, were not significantly different between the two groups.

The elderly population are the most likely to develop hip fractures [13]. China has the largest elderly population, with a rapidly ageing population, and faces the challenge of an increase in cases developing hip fractures. An epidemiological study in mainland China [14] showed that the incidence of hip fractures in patients aged ≥ 55 years and older was 136.65 per 100,000 in 2016; however, the absolute hip fracture cases and hospitalisation costs increased rapidly. Currently, there are few studies on hip fractures in the oldest-old population. A multicentre retrospective study [15] on centenarians with hip fractures conducted in the UK revealed that prompt surgical treatment is essential to restore functions in patients suitable for surgery. Furthermore, although mortality within one year of injury is high, approximately 50% of the patients survive for > 1 year after injury. According to Wang et al. [16], postoperative respiratory failure is an independent risk factor for mortality in the oldest-old patients. Kim et al. [17] demonstrated a higher mortality rate in nonagenarians with hip fractures. Nonagenarians with hip fractures were more likely to die if their ASA scores were higher and the time between trauma and surgery was longer. Simultaneously, nonagenarians with hip fractures maintained a lower rate of preinjury walking ability. Furthermore, integrated bundle management may benefit the oldest-old patients, according to Fu et al. [9].

In recent years, several studies, including prospective multicentre studies with a high level of evidence-based medicine, have reported optimal anaesthesia for patients with hip fractures. According to Zhang et al. [6], GA and NA may result in different blood loss rates. A systematic review [18] of 15 studies demonstrated shorter hospital stays in patients with hip fractures treated under NA; however, higher-quality studies with larger sample sizes are required to clarify this. A study on the guide to managing hip fractures in the UK conducted in 2020 [19] suggested that different types of anaesthesia may have less impact on patient outcomes and that careful implementation of the anaesthesia technique may be more important than the type of anaesthesia used. Different anaesthetic management methods for hip fracture surgery are associated with different patient outcomes. For example, intraoperative hypotension is progressively associated with a significant increase in mortality at 5 and 30 days postoperatively [20]. A randomised controlled trial by Neuman et al. [21] revealed that NA is associated with more pain in the first 24 h after surgery.

In this study, mortality between the two groups within 30 days after surgery did not show a statistically significant difference. Furthermore, the relationship between anaesthesia and postoperative mortality in several studies is controversial. A retrospective study conducted in Taiwan [22] showed that using the NA technique in patients with hip fractures was associated with a lower risk of in-hospital mortality. In addition, patients who underwent surgery with NA had lower costs, ICU admission rates, and length of stay. However, a study by Gilbert et al. [23] showed no significant difference in postoperative mortality among elderly patients with hip fractures treated under different anaesthesia techniques. A retrospective study by Neuman et al. [24] revealed that regional anaesthesia was not associated with a lower 30-day mortality rate but with a modestly shorter hospital stay than GA. Therefore, conducting further studies is necessary.

Acute myocardial infarction, heart failure, and stroke were not significantly different between the two anaesthesia methods in this study. Cardio-cerebral vascular complications tended to be associated with hemodynamic disturbances in patients. In our centre, we inferred that it is crucial to ensure intraoperative hemodynamic stability regardless of the anaesthetic method used. However, both anaesthesia methods can alter the intraoperative haemodynamics of patients [25]. Previous studies have suggested that insertion and removal of endotracheal tubes during GA in elderly patients leads to hemodynamic instability [26]. For patients receiving spinal-epidural anaesthesia, NA causes sympathetic inhibition and vagal hyperactivity, resulting in peripheral vasodilation, hypotension, and decreased heart rate [27]. Wang et al. [16] concluded that the interval between the incidence of fractures and surgeries may lead to excessive blood loss and fatal diseases such as cerebrovascular disease.

This study found no statistically significant difference between the incidence of postoperative pneumonia and the anaesthesia type in the oldest-old patients with IFs. Several studies [6, 28, 29] have reported no significant difference in the incidence of postoperative pneumonia between the two anaesthesia types in patients with hip fractures. Studies [30] have shown that age is associated with changes in lung system immunity. Dysregulation of pro-inflammatory mediators and antimicrobial defence systems reduces lung function and its response to infection. Therefore, ageing increases the risk of postoperative pulmonary complications due to loss of physiological reserve and airway defence. Thus, respiratory system-related complications should be a primary concern in oldest-old patients after surgery. Wang et al. [16] suggested that postoperative respiratory failure was an independent risk factor for mortality in nonagenarians and centenarians. Therefore, in our centre, we inferred that good airway management is crucial regardless of the anaesthetic method used.

In this study, patients in the NA group (15.07%) had a lower probability of deep venous thrombosis than the GA group (24.07%), but the difference between the two groups was not significant. Previous studies concluded that NA is associated with a reduction in deep venous thrombotic events compared with GA [6]. In NA, the sympathetic blockade can lead to lower extremity vasodilatation, and increased blood flow in the lower extremity may reduce the coagulability and viscosity of blood, increasing the probability of deep venous thrombotic events [31].

Delirium is a critical postoperative adverse event that can lead to long-term cognitive and functional decline, prolonged hospital stays, and increased difficulty of postoperative rehabilitation. In this study, the incidence of postoperative delirium in patients receiving NA was significantly lower than that in patients receiving GA, and statistically significant differences were observed between them. Controversies exist regarding the effect of anaesthetic methods on delirium in recent studies. Studies have demonstrated the benefits of postoperative cognitive function when intubation is used in spinal-epidural anaesthesia [5]; this may be related to residual depression of the central nervous system by systemic analgesics in patients undergoing GA with intubation [32]. The European Society of Anesthesiology consensus guidelines on postoperative delirium recommend that benzodiazepines should be avoided preoperatively in elderly patients, and anaesthesia depth should be monitored to avoid postoperative delirium [33]. If NA requires sedation, dexmedetomidine may be used. There have been previous meta-analyses showing that dexmedetomidine is effective at reducing surgery-induced delirium [34]. However, a randomised controlled trial [35] involving 950 Chinese patients revealed no statistically significant difference in the incidence of postoperative delirium in patients with hip fractures receiving NA or GA, and the type of anaesthesia did not affect the incidence of postoperative delirium.

In this study, there were no significant differences between the two groups regarding postoperative electrolyte imbalance, hypoproteinaemia, or anaemia. Aging can lead to decreased physiological function of many organs, especially in the oldest-old population [36]. When the digestive system function declines, the absorption function of the human body and the synthesis of nutrients are affected. After trauma, elderly patients develop a severe stress response that activates the hypothalamic-pituitary-adrenocortical axis, releasing catecholamines and stress hormones into the blood [37]. Catecholamines and stress-related hormones can put the body in a hypermetabolic state, leading to increased blood glucose and protein and lipid catabolism [38]. The combined effect of these factors with stress-induced eating reduction will cause postoperative hypohaemoglobin, hypoalbumin, hyponatraemia, hypokalaemia, and other complications, critically affecting the patient. Serum albumin levels are indicators of nutritional status. Albumin is a negatively charged substance synthesised by the liver and is an acute-phase reactant [39]. Previous studies have suggested that low albumin levels are associated with increased mortality from hip fractures [40]. Therefore, nutritional support should be actively provided to patients at nutritional risk. Nutritional support can help correct hypoproteinaemia and maintain fluid, electrolyte, and acid-base balance.

This study has several limitations. First, it was a retrospective study with a small sample size. Randomised controlled trials with larger patient samples are required for a more detailed comparison of the two anaesthesia methods. This study only investigated the effects of different anaesthesia types in the oldest-old patients with IF treated using PFNA implantation. Further studies are needed for oldest-old patients with femoral neck fractures or those treated with other surgical techniques. Nevertheless, this study reports cumulative mortality and requires subsequent risk factor analyses for postoperative adverse events.

## Conclusion

There is no evidence to suggest that the anaesthesia type affects heart failure, acute stroke, acute myocardial infarction, pulmonary disease, anaemia, deep vein thrombosis, hypoproteinaemia, electrolyte disorders, or death within 30 days after surgery in oldest-old patients with IFs. However, NA may be associated with a lower incidence of postoperative delirium.

## Data Availability

The data used and analysed in this study are included in the article or are available from the corresponding first authors upon reasonable request.

## Abbreviations

ASA: American Society of Anesthesiologists
BMI: Body mass index
GA: General anaesthesia
ICU: Intensive care unit
IF: Intertrochanteric fractures
NA: Neuraxial anaesthesia
PFNA: Proximal femoral nail antirotation
